# Antioxidant and Hypolipidemic Activities of Cinnamic Acid Derivatives

**DOI:** 10.3390/molecules28186732

**Published:** 2023-09-21

**Authors:** Christina Nouni, Panagiotis Theodosis-Nobelos, Eleni A. Rekka

**Affiliations:** 1 Department of Pharmaceutical Chemistry, School of Pharmacy, Aristotelian University of Thessaloniki, 54124 Thessaloniki, Greece; 2 Department of Pharmacy, School of Health Sciences, Frederick University, Nicosia 1036, Cyprus; hsc.np@frederick.ac.cy

**Keywords:** cinnamic acid derivatives, morpholine, 3-phenylacrylic acid, antioxidants, hyperlipidemia, hypolipidemic agents

## Abstract

Oxidative stress and hyperlipidemia are important factors for the initiation and progression of various cell degenerative pathological conditions, including cardiovascular and neurological diseases. A series of cinnamic acid-derived acids, such as ferulic acid, sinapic acid, 3,4-dimethoxycinnamic acid, *p*-coumaric acid, and (*E*)-3-(3,5-di-*tert*-butyl-4-hydroxyphenyl)acrylic acid, were esterified or amidated with various moieties, bearing different biological activities, and evaluated. The antioxidant and radical scavenging abilities of the compounds via inhibition of rat hepatic microsomal membrane lipid peroxidation, as well as their interaction with the stable radical 2,2-diphenyl-1-picrylhydrazyl (DPPH), were assessed. Further, their hypolipidemic activity in vivo was tested. The majority of the obtained compounds demonstrated considerable radical scavenging and antioxidant action, with a parallel decrease in Triton-induced hyperlipidemia in rats. The (*E*)-3-(3,5-di-*tert*-butyl-4-hydroxyphenyl)acrylic acid derivative with morpholine and 4-methylpiperidine (compounds **4** and **13**, respectively) significantly decreased triglycerides and total cholesterol in the plasma of hyperlipidemic rats, with an antioxidant capacity similar to that of the antioxidant Trolox. The compounds were designed to exhibit antioxidant and hypolipidemic pharmacological actions, and this succeeded for the majority of them. Thus, such agents may be of interest in conditions and diseases implicating oxidative stress and dyslipidemia.

## 1. Introduction

Reactive oxygen and nitrogen species (ROS and RNS) contribute considerably to the pathological mechanisms of cardiovascular and nervous system diseases, like atherosclerosis, metabolic disturbances, and neurodegenerative disorders [1]. Hyperlipidemia is involved in endothelial ROS production. Thus, oxidative stress and hyperlipidemia are related to the promotion of atherogenesis, indicating their additive consequences in atherosclerotic incidents [2]. Such effects are intensified in diets rich in saturated lipids, which may lead to metabolic syndrome and disturbed cognitive processes, with increased lipid peroxidation levels [3,4].

Cinnamic acid and its analogues, like cinnamaldehyde, have anti-inflammatory activity and suppress, directly and/or indirectly, oxidative stress, modulating cyclooxygenase-2, inducible NO synthase, and NF-KB levels [5]. Ferulic acid is a bioactive antioxidant with inhibitory activity on platelet aggregation, liver cholesterol synthesis, and LDL-cholesterol oxidation [6], whilst sinapic acid decreases LDL peroxidation induced by peroxynitrate [7]. In addition, ferulic acids and various methylated cinnamic acid derivatives, like sinapic acid and 3,4-dimethoxycinnamic acid, improve cell viability and indirectly increase the expression of various endogenous antioxidant enzymes [8]. Finally, *p*-coumaric acid (4-hydroxycinnamic acid) [9], as well as derivatives of (*E*)-3-(3,5-di-*tert*-butyl-4-hydroxyphenyl)acrylic acid [10], possess antioxidant, hypolipidemic, and anti-inflammatory activity. However, apart from their positive characteristics, some toxicity and interaction issues arise. The majority of them bind with albumin, a fact that may be able to provoke interactions at the distribution level [11]. Additionally, some of them may exert their pro-oxidant effect in the presence of labile metals, causing DNA damage and inhibiting cell proliferation [12], whilst ferulic acid, cinnamic acid, and caffeic acid have shown clastogenic effects in rat hepatoma tissue cells [13].

**Figure 1:**
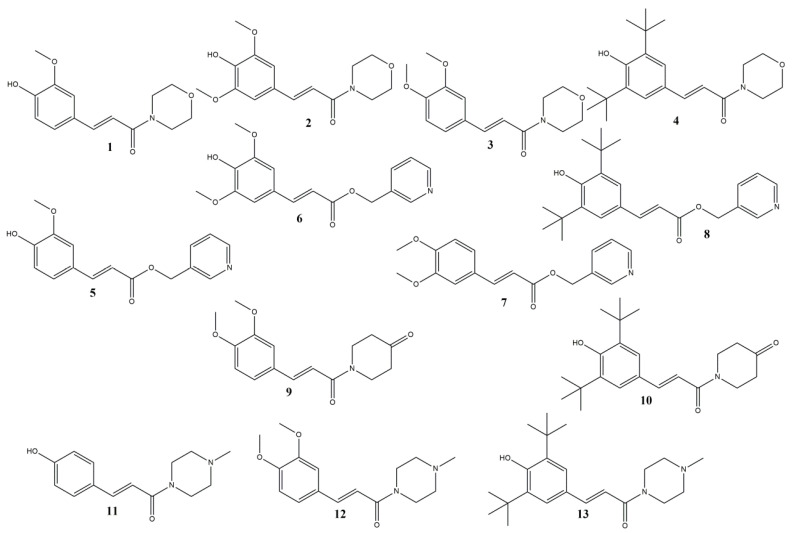
Structures of the synthesized compounds.

Considering the above evidence, in this investigation, ferulic acid, sinapic acid, 3,4-dimethoxycinnamic acid, p-coumaric acid, and (*E*)-3-(3,5-di-*tert*-butyl-4-hydroxyphenyl)acrylic acid were used. These acids were amidated with morpholine since it was previously found that a number of 2,4-substituted morpholines [14] and thiomorpholines [15] possess considerable hypolipidemic and antioxidant activities. Furthermore, amides of non-steroidal anti-inflammatory drugs, with unsubstituted thiomorpholine, are potent hypolipidemic and anti-inflammatory compounds [16]. Then, the morpholine oxygen was replaced by either C=O (the oxygen removed outside the heterocyclic ring) or N-CH_3_ (Figure 1) in order to compare their potential activities and make further structure–activity relationship deductions.

Moreover, piperidine and piperazine consist of the main parts of various approved drugs like CNS (central nervous system) active donepezil, haloperidol, and risperidone, as well as anti-anginal trimetazidine and ranolazine, with antioxidant, hypolipidemic, and antimicrobial characteristics [17,18,19]. Four of our five starting acids were also esterified with 3-hydroxymethylpyridine (nicotinyl alcohol), a molecule that, among other effects, has shown hypolipidemic potency and a reduction in the extent and severity of myocardial ischemic injury [20].

The aim of this work was to investigate whether such derivatives would combine hypolipidemic, antioxidant, and radical scavenging activities in order to affect, with one molecule, in a multi-targeting way, diseases with a pleiotropic range of pathology that may require multi-drug treatment. Thus, with the combination, in one molecule, of moieties with potential biological activities, we strived towards this direction. The antioxidant activity of the compounds was evaluated as inhibition of rat hepatic microsomal membrane lipid peroxidation and as interaction with the stable radical 2,2-diphenyl-1-picrylhydrazyl (DPPH). The in vivo hypolipidemic activity of the compounds was estimated in Triton-induced hyperlipidemic rats, as well as plasma total cholesterol and triglyceride levels.

## 2. Results and Discussion

### 2.1. Synthesis

**Figure 2:**
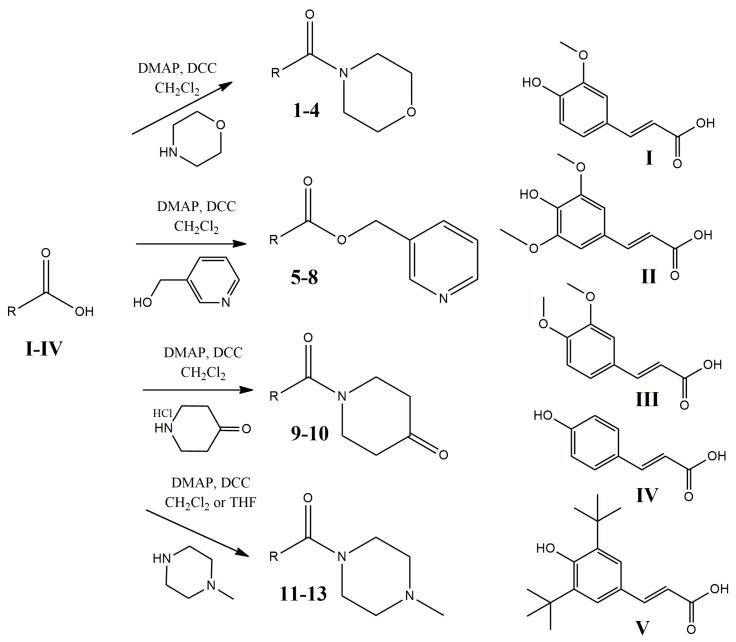
Synthesis of the examined compounds **1**–**13**. The corresponding acids (**I**–**V**) were activated with DCC and with the corresponding alcohol or amine. In the presence of DMAP, the esterification or amidation reaction was carried out, providing the corresponding **1**–**13** compounds.

Compounds **1**–**13** were synthesized by direct amidation or esterification, with morpholine, 3-hydroxymethylpyridine, 4-piperidone, and 1-methylpiperazine of the carboxylic group of the respective acids **I**–**IV**, using *N*,*N*′-dicyclohexylcarbodiimide (DCC) and 4-dimethylaminopyridine (DMAP), at room temperature (Figure 2). The overall yields were satisfactory, in the range of 61–82%. The final compounds were purified by flash column chromatography. In the case of amine derivatives **5**–**8** and **11**–**13**, triethylamine was added to the eluting solution.

DMAP: 4-dimethylaminopyridine; DCC: *N,N*’-dicyclohexylcarbodiimide; THF: tetrahydrofuran;
**I**: 3-(4-hydroxy-3-methoxyphenyl)acrylic acid; **II**: 3-(4-hydroxy-3,5-dimethoxyphenyl)acrylic acid;
**III**: 3-(3,4-dimethoxyphenyl)acrylic acid; **IV**: 3-(4-hydroxyphenyl)acrylic acid;
**V**: 3-(3,5-di-*tert*-butyl-4-hydroxyphenyl)acrylic acid;
compound **1**: (*E*)-3-(4-hydroxy-3-methoxyphenyl)-1-morpholinoprop-2-en-1-one;
compound **2**: (*E*)-3-(4-hydroxy-3,5-dimethoxyphenyl)-1-morpholinoprop-2-en-1-one;
compound **3**: (*E*)-3-(3,4-dimethoxyphenyl)-1-morpholinoprop-2-en-1-one;
compound **4**: (*E,Z*)-3-(3,5-di-*tert*-butyl-4-hydroxyphenyl)-1-morpholinoprop-2-en-1-one;
compound **5**: (*E*)-pyridin-3-ylmethyl 3-(4-hydroxy-3-methoxyphenyl)acrylate;
compound **6**: (*E*)-pyridin-3-ylmethyl 3-(4-hydroxy-3,5-dimethoxyphenyl)acrylate;
compound **7**: (*E*)-pyridin-3-ylmethyl 3-(3,4-dimethoxyphenyl)acrylate;
compound **8**: (*E*)-pyridin-3-ylmethyl 3-(3,5-di-*tert*-butyl-4-hydroxyphenyl)acrylate;
compound **9**: (*E*)-1-(3-(3,4-dimethoxyphenyl)acryloyl)piperidin-4-one;
compound **10**: (*E*)-1-(3-(3,5-di-*tert*-butyl-4-hydroxyphenyl)acryloyl)piperidin-4-one;
compound **11**: (*E*)-3-(4-hydroxyphenyl)-1-(4-methylpiperazin-1-yl)prop-2-en-1-one;
compound **12**: (*E*)-3-(3,4-dimethoxyphenyl)-1-(4-methylpiperazin-1-yl)prop-2-en-1-one;
compound **13**: (*E*)-3-(3,5-di-*tert*-butyl-4-hydroxyphenyl)-1-(4-methylpiperazin-1-yl)prop-2-en-1-one

### 2.2. Biological Evaluation

#### 2.2.1. Effect on Lipid Peroxidation

Reactive oxygen and nitrogen species are an integral part of the initiation and progression of vascular, neuronal, and lipid homeostasis disturbance via alteration in lipid bilayer, protein, sugar and DNA oxidation, and mitochondrial dysfunction [21,22]. Τhe synthesized compounds were tested for their antioxidant activity, expressed as inhibition of rat microsomal membrane lipid peroxidation induced by ferrous ascorbate. The IC_50_ values of the active final compounds **4**, **5**, **6**, **8**, **9**, **10**, **13**, and **Trolox**, a well-known antioxidant, used as reference after 45 min of incubation, are shown in Table 1.

Phenols, in general, act as chain-breaking antioxidants, as the phenolic hydrogen atom can be transferred to a radical, while the remaining phenoxyl radical can be stabilized by resonance. Thus, the non-phenolic compounds **3**, **7**, and **12** were inactive. Interestingly, compound **9** seems to be a weak antioxidant. This could partly be explained by the observation that piperidone moiety may act as a Lewis base for Fe^2+^ complexation [23]. However, there are different factors influencing the degree of antioxidant potency of phenols, such as bond dissociation enthalpy (BDE) of the O-H bond and the degree of ionization of phenols [24]. The above are obviously related to the nature of substitution on the phenolic ring. Low dissociation enthalpy favors phenolic hydrogen atom abstraction, and high ionization impedes this transfer [24]. According to these criteria, 2,6-di-*tert*-butyl substitution is the most privileged for antioxidant activity, with low BDE and ionization. No *ortho*-substitution on the phenol ring is the least favored structure. In between are the 2,6-dimethoxy derivatives, followed by the 2-methoxy compounds. Indeed, **4**, **8**, **10**, and **13** are the most potent antioxidants, with very similar IC_50_ values, independent of the heterocyclic moieties and comparable to Trolox, an effect that is in accordance with previous results relevant to this moiety [25,26]. Compounds **6** and **5** were the next two most active compounds concerning antioxidant activity. Compound **6** was more active, perhaps due to the electron-donating activity of the extra methoxy group. The lack of adjacent electron-donating groups may also explain the lack of activity of compound **11**. Compounds **1** and **2**, unexpectedly, do not possess any activity despite their expected molecular characteristics. Possibly, low lipophilicity due to the morpholine ring renders them unable to approach the lipid phase, the site of lipid peroxidation, efficiently. On the other hand, unexpectedly, compound **9**, with no obvious hydrogen abstraction characteristics, offered moderate lipid peroxidation inhibitory activity, a result that may at least partially be derived from the labille hydrogen atom adjacent to the ketone group of piperidin-4-one.

#### 2.2.2. Interaction with DPPH

The interaction of compounds with the lipophilic, electron-accepting, *N*-centred 1,1-diphenyl-2-picrylhydrazyl (DPPH) stable free radical is a common antioxidant assay and a method for the evaluation of the reducing activity of the compounds. The interaction of the active compounds **2**, **4**, **5**, **6**, **8**, **10**, and **13** at various concentrations with DPPH is provided in Table 2.

The activity of the compounds in this experiment was similar to the lipid peroxidation inhibitory potency, with the exception of compound **9**, which was inactive, and compound **2**, which seems to be of equal activity with the most active compounds in both experiments, e.g., compounds **4**, **8**, **10**, and **13**. This effect may be related to the better dissolution of compound **2** in the ethanolic mixture. In a similar manner, compounds **5** and **6** were less active than the other drastic derivatives. The lack of free phenolic hydrogen atoms may be the main reason for the inactivity of compound **9**. The di-*tert*-butyl moiety may be one of the main causes of such an increased reduction in DPPH radicals of compounds **4**, **8**, **10**, and **13**, whilst the dimethoxy substitution of compounds **2** and **6** seems to follow as the main factor for the hydrogen-donating effect. Monomethoxy substitution seems to offer lower (compound **5**) and, occasionally (compound **1**), zero additive-reducing effect. Furthermore, if we take into account the effect of Trolox in this experiment, we can realize that our active compounds were of similar activity, or more active, than Trolox, especially the di-*tert*-butyl moiety containing compounds **4**, **8**, **10**, and **13**.

#### 2.2.3. Effect of Compounds on Hyperlipidemia in Rats

Hyperlipidemia is correlated with the propagation of oxidative stress and inflammatory conditions in cardiovascular and central nervous system disorders [21,27].

The effect of compounds **1**–**13** under investigation on plasma total cholesterol and triglycerides, 24 h post-injection, determined after Triton-induced hyperlipidemia in rats, is shown in Table 3. Simvastatin is included for comparison.

Tyloxapol (Triton WR133) is a nonionic surfactant used to induce hyperlipidemia in experimental animals after parenteral administration with maximal concentration of triglycerides, VLDL- and LDL-cholesterol, in plasma, after 24 h, mainly due to inhibition of lipoprotein lipase and stimulation of 3-hydroxy-3-methyl-glutaryl-CoA (HMG-CoA) reductase [28,29].

Compounds **1–13** were administered at 0.15 mmol/kg and reduced total cholesterol and triglycerides statistically significantly (with the exception of compound **1**), in most cases extremely significantly, with compounds **4**, **6**, and **11**–**13** being the most active. For all the compounds, the animals appeared normal 24 h post administration, both macroscopically and by autopsy. For compounds **4**, **6**, and **13**, these results may, at least partly, be related to their antioxidant effect. Concerning the morpholine ring, it seems that the most successful structural change was the replacement of this ring with *N*-methyl-piperazine residue. It can be realized that this structural element contributes to the hypolipidemic activity of compounds **11** and **12**, which lack antioxidant action, and amplifies the effect of the antioxidant molecules, i.e., compound **13** vs. **4**, **8**, and **10**.

Finally, an important finding in the present investigation is that the synthesized compounds cause a significant reduction in triglycerides, concomitantly with cholesterol decrease. It is known that there is still a risk for atherosclerotic cardiovascular disease in patients with hypertriglyceridemia, despite reduction in cholesterol levels with statins [30].

## 3. Materials and Methods

### 3.1. General Methods

All commercially available reagents were purchased from Merck (Kenilworth, NJ, USA) or Sigma (St. Louis, MO, USA) (dichloromethane, tetrahydrofuran, petroleum ether, ethyl acetate, ethanol absolute, dimethylsulfoxide, 4-dimethylaminopyridine, *N,N*’-dicyclohexylcarbodiimide, morpholine, 3-hydroxymethylpyridine, 4-piperidone hydrochloride, 1-methylpiperazine, *p*-coumaric acid, ferulic acid, sinapic acid, 3,4-dimethoxycinnamic acid, 3-(3,5-di-*tert*-butyl-4-hydroxyphenyl)acrylic acid, concentrated HCl, NaHCO_3_, NaCl, Na_2_SO_4_, Κ_2_CO_3_, triethylamine, ascorbic acid, Tris–HCl/KCl, FeSO_4_, Tween 80, tyloxapol, 1,1-diphenyl-2-picrylhydrazyl) and used without further purification. The IR spectra were recorded on a Perkin Elmer Spectrum BX FT-IR (Waltham, MA, USA) spectrometer. The ^1^H NMR spectra were recorded using an AGILENT DD2-500 MHz (Santa Clara, CA, USA) spectrometer. All chemical shifts are reported in *δ* (ppm), and signals are provided as follows: s, singlet; d, doublet; t, triplet; m, multiplet. Melting points (m.p.) were determined with a MEL-TEMPII (Laboratory Devices, Sigma-Aldrich, Milwaukee, WI, USA) apparatus and were uncorrected. The microanalyses were performed on a Perkin-Elmer 2400 CHN elemental analyzer (Waltham, MA, USA). Thin-layer chromatography (TLC silica gel 60 F254 aluminum sheets, Merck (Kenilworth, NJ, USA)) was used to follow the reactions, and the spots were visualized under UV light. UV-visible determinations were performed using a Shimadzu UV-1700 Pharma Spec spectrophotometer (Kyoto, Japan). For the determination of plasma cholesterol and triglyceride concentrations, SPINREACT S.A. Cholesterol-LQ and Triglycerides-LQ kits were used (Ctra. Santa Coloma, Spain).

Wistar male rats (160–220 g, 3–4 months old) were kept in the Centre of the School of Veterinary Medicine (EL54 BIO42), Aristotelian University of Thessaloniki, which is registered by the official state veterinary authorities (presidential degree 56/2013, in harmonization with the European Directive 2010/63/EEC). The experimental protocols were approved by the Animal Ethics Committee of the Prefecture of Central Macedonia (no. 270079/2500).

### 3.2. Synthesis

#### 3.2.1. General Method for the Synthesis of Compounds **1**–**4**

In a solution of the corresponding acid (1 mmol) in dichloromethane (CH_2_Cl_2_), 4-dimethylaminopyridine (DMAP, 0.5 mmol), morpholine (1 mmol), and *N,N*’-dicyclohexylcarbodiimide (DCC, 1.2 mmol) were added. The reaction mixture was stirred for 24 h at room temperature under nitrogen. After filtration, the solution was washed with aqueous HCl 5% solution, aqueous NaHCO_3_ 5% solution, and saturated NaCl solution and dried over Na_2_SO_4_. The final compounds were isolated from the mixture with flash chromatography using petroleum ether and ethyl acetate as eluents.

*(E)-3-(4-hydroxy-3-methoxyphenyl)-1-morpholinoprop-2-en-1-one,* **1**. 

Flash chromatography (petroleum ether/ethyl acetate, 1/1). White solid, yield 71%, m.p. 89–92 °C. IR (nujol): 3400–3200 (O-H), 1644 (C=O amide), 1587 (C-C aromatic), and 1038 (C-O morpholine) cm^−1^. ^1^H NMR (CDCl_3_) *δ*: 3.76–3.68 (m, 8H, -C**H_2_**^−^ morpholine), 3.93 (s, 3H, C**H_3_**O-), 5.90 (s, 1H -OH), 6.68 (d, *J* = 15.3 Hz, 1H ArHC=C**H**-), 6.92 (d, 1H, *J* = 8.2 Hz, aromatic **H4**), 7.00 (d, 1H, *J* = 1.8 Hz, aromatic **H2**), 7.10 (dd, 1H, *J* = 8.2, 1.8 Hz, aromatic **H6**), and 7.66 (d, 1H, *J* = 15.3 Hz, Ar**H**C=CH-) [31,32,33].

*(E)-3-(4-hydroxy-3,5-dimethoxyphenyl)-1-morpholinoprop-2-en-1-one,* **2**. 

Flash chromatography (petroleum ether/ethyl acetate, 1/1). White solid, yield 75%, m.p. 191–194 °C. IR (nujol): 3400–3200 (O-H), 1648 (C=O amide), 1599, 1584 (C-C aromatic), and 1038 (C-O morpholine) cm^−1^. ^1^H NMR (CDCl_3_) *δ*: 3.75 (m, 8H, -C**H_2_**^−^ morpholine), 3.92 (s, 6H C**H_3_**O-), 5.76 (s, 1H -O**H**), 6.67 (d, 1H, *J* = 15.3 Hz, ArHC=C**H**-), 6.75 (s, 2H, aromatic **H2**, **H4**), and 7.61 (d, 1H, *J* = 15.3 Hz, Ar**H**C=CH-) [34].

*(E)-3-(3,4-dimethoxyphenyl)-1-morpholinoprop-2-en-1-one,* **3**.

Flash chromatography (petroleum ether/ethyl acetate, 2/1). White solid, yield 72%, m.p. 122–124 °C. IR (nujol): 1650 (C=O amide), 1592 (C-C aromatic), and 1038 (C-O morpholine) cm^−1^. ^1^H NMR (CDCl_3_) *δ*: 3.74 (dd, 8H, *J* = 20.8, 3.4 Hz, -C**H_2_**^−^ morpholine), 3.91, 3.90 (s, 6H, C**H_3_**O-), 6.69 (d, 1H, *J* = 15.3 Hz, ArHC=C**H**-), 6.85 (d, 1H, *J* = 8.3 Hz, aromatic **H6**), 7.02 (d, 1H, *J* = 1.8 Hz, aromatic **H2**), 7.10 (dd, 1H, *J* = 8.3, 1.9 Hz, aromatic **H5**), and 7.64 (d, 1H, *J* = 15.3 Hz, Ar**H**C=CH-) [35,36,37].

*(E,Z)-3-(3,5-di-tert-butyl-4-hydroxyphenyl)-1-morpholinoprop-2-en-1-one,* **4**.

Flash chromatography (petroleum ether/ethyl acetate, 5/1). White solid, yield 80%, m.p. 72–74 °C. IR (nujol): 3355 (O-H), 1644 (C=O amide), 1592 (C-C aromatic), and 1038 (C-O morpholine) cm^−1^. ^1^H NMR (CDCl_3_) *δ*: 1.45 (s, 18H, (C**H_3_**)_3_C-), 3.90–3.58 (m, 8H, -C**H_2_**- morpholine), 5.55, 5,45 (s, 1H –O**H**), 6.31 (d, 1H, *J* = 15.9 Hz, ArHC=C**H**- trans), 6.64 (d, 1H, *J* = 15.3 Hz, ArHC=C**H**- cis), 7.39, 7.35 (s, 2H, aromatic **H1** and **H6**), 7.67 (d, 1H, *J* = 15.3 Hz, Ar**H**C=CH- cis), and 7.74(d, 1H, *J* = 15.9 Hz, Ar**H**C=CH- trans). Anal. Calcd for C_21_H_31_ΝO_3_: C, 73.01; H, 9.04; Ν, 4.05. Found: C, 72.86; H, 8.97; Ν, 4.20.

#### 3.2.2. General Method for the Synthesis of Compounds **5**–**8**

In a solution of the corresponding acid (1 mmol) in dichloromethane (CH_2_Cl_2_), 4-dimethylaminopyridine (DMAP, 0.5 mmol), 3-hydroxymethylpyridine (1 mmol), and *N,N*’-dicyclohexylcarbodiimide (DCC, 1.2 mmol) were added. The reaction mixture was stirred for 24 h at room temperature under nitrogen. After filtration, the solution was washed with aqueous NaHCO_3_ 5% solution, saturated NaCl solution, and dried over Κ_2_CO_3_. The final compounds were isolated with flash chromatography using petroleum ether and ethyl acetate as eluents containing triethylamine 0.04% *v*/*v*.

*(E)-pyridin-3-ylmethyl 3-(4-hydroxy-3-methoxyphenyl)acrylate,* **5**. 

Flash chromatography (petroleum ether/ethyl acetate, 1/1). White solid, yield 71%, m.p. 168 °C. IR (nujol): 3400–3200 (O-H), 1695 (C=O ester), and 1583 (C-C aromatic) cm^−1^. ^1^H NMR (CDCl_3_) *δ*: 3.92 (s, 3H, C**H_3_**O-), 5.26 (s, 2H, -OC**H_2_**^−^), 5.95 (s, 1H, -O**H**), 6.32 (d, 1H, *J* = 15.9 Hz, ArHC=C**H**-), 6.92 (d, 1H, *J* = 8.2 Hz, aromatic phenyl **H5**), 7.03 (d, 1H, *J* = 1.8 Hz, aromatic phenyl **H1**), 7.08 (dd, 1H, *J* = 8.2, 1.8 Hz, aromatic phenyl **H6**), 7.32 (dd, 1H, *J* = 7.8, 4.8 Hz, pyridine **H5**), 7.66 (d, 1H, *J* = 15.9 Hz, Ar**H**C=CH-), 7.75 (dd, 1H, *J* = 7.8, 1.7 Hz, pyridine **H4**), 8.59 (dd, 1H, *J* = 4.8, 1.4 Hz, pyridine **H6**), and 8.69 (d, 1H, *J* = 1.8 Hz, pyridine **H2**). Anal. Calcd for C_16_H_15_ΝO_4_: C, 67.36; H, 5.30; Ν, 4.91. Found: C, 67.21; H, 5.34; Ν, 4.79.

*(E)-pyridin-3-ylmethyl 3-(4-hydroxy-3,5-dimethoxyphenyl)acrylate,* **6**. 

Flash chromatography (petroleum ether/ethyl acetate, 1/2). White solid, yield 65%, m.p. 165 °C. IR (nujol): 3400–3200 (O-H), 1696 (C=O ester) and 1583 (C-C aromatic) cm^−1^. ^1^H NMR (CDCl_3_) *δ*: 2.17 (s, 1H, -O**H**), 3.91 (s, 6H, C**H_3_**O-), 5.26 (s, 2H -OC**H_2_**-), 6.33 (d, 1H, *J* = 15.9 Hz, ArHC=C**H**-), 6.77 (s, 2H, aromatic phenyl **H1** and **H6**), 7.34 (dd, 1H, *J* = 7.2, 5.1 Hz, pyridine **H5**), 7.64 (d, 1H, *J* = 15.9 Hz, Ar**H**C=CH-), 7.76 (d, 1H, *J* = 7.8 Hz, pyridine **H4**), 8.61 (s, 1H, pyridine **H6**), and 8.71 (s, 1H, pyridine **H2**). Anal. Calcd for C_17_H_17_ΝO_5_: C, 64.75; H, 5.43; Ν, 4.44. Found: C, 64.81; H, 5.47; Ν, 4.52.

*(E)-pyridin-3-ylmethyl 3-(3,4-dimethoxyphenyl)acrylate,* **7**. 

Flash chromatography (petroleum ether/ethyl acetate, 1/1). White solid, yield 74%, m.p. 85 °C. IR (nujol): 1710 (C=O ester), 1595, and 1580 (C-C aromatic) cm^−1^. ^1^H NMR (CDCl_3_) *δ*: 3.91, 3.90 (s, 6H, C**H_3_**O-), 5.26 (s, 2H, -OC**H_2_**-), 6.34 (d, 1H, *J* = 15.9 Hz, ArHC=C**H**-), 6.86 (d, 1H, *J* = 8.3 Hz, aromatic phenyl **H5**), 7.04 (d, 1H, *J* = 1.9 Hz, aromatic phenyl **H2**), 7.10 (dd, 1H, *J* = 8.3, 1.9 Hz, aromatic phenyl **H6**), 7.32 (dd, 1H, *J* = 7.8, 4.8 Hz, pyridine **H5**), 7.67 (d, 1H, *J* = 15.9 Hz, Ar**H**C=CH-), 7.78–7.73 (m, 1H, pyridine **H4**), 8.59 (dd, 1H, *J* = 4.8, 1.2 Hz, pyridine **H6**), and 8.69 (d, 1H, *J* = 1.2 Hz, pyridine **H2**). Anal. Calcd for C_17_H_17_ΝO_4_: C, 68.21; H, 5.72; Ν, 4.68. Found: C, 68.23; H, 5.63; Ν, 4.59.

*(E)-pyridin-3-ylmethyl 3-(3,5-di-tert-butyl-4-hydroxyphenyl)acrylate,* **8**. 

Flash chromatography (petroleum ether/ethyl acetate, 4/1). White solid, yield 69%, m.p. 172 °C. IR (nujol): 3418 (O-H), 1710 (C=O ester), 1595, and 1581 (C-C aromatic) cm^−1^. ^1^H NMR (CDCl_3_) *δ*: 1.44 (d, 18H, *J* = 4.6 Hz, (C**H_3_**)**_3_**C-), 5.26 (s, 2H, -OC**H_2_**-), 5.54 (s, 1H, -O**H**), 6.32 (d, 1H, *J* = 15.9 Hz, ArHC=C**H**-), 7.33 (dd, 1H, *J* = 7.8, 4.9 Hz, pyridine **H5**), 7.36 (s, 2H, aromatic phenyl **H1** and **H6**), 7.69 (d, 1H, *J* = 15.9 Hz, Ar**H**C=CH-), 7.77 (d, 1H, *J* = 7.8 Hz, pyridine **H4**), 8.61–8.58 (m, 1H, pyridine **H6**), and 8.69 (s, 1H, pyridine **H2**). Anal. Calcd for C_23_H_29_ΝO_3_: C, 75.17; H, 5.43; Ν, 4.44. Found: C, 75.23; H, 5.38; Ν, 4.61.

#### 3.2.3. General Method for the Synthesis of Compounds **9**–**10**

In a solution of the corresponding acid (1 mmol) in dichloromethane (CH_2_Cl_2_), 4-dimethylaminopyridine (DMAP, 1.5 mmol), 4-piperidone hydrochloride (1 mmol), and *N,N*’-dicyclohexylcarbodiimide (DCC, 1.2 mmol) were added. The reaction mixture was stirred for 24 h at room temperature under nitrogen. After filtration, the solution was washed with aqueous NaHCO_3_ 5% solution, saturated NaCl solution, and dried over Na_2_SO_4_. The final compounds were isolated with flash chromatography using petroleum ether and ethyl acetate as eluents.

*(E)-1-(3-(3,4-dimethoxyphenyl)acryloyl)piperidin-4-one,* **9**. 

Flash chromatography (petroleum ether/ethyl acetate, 1/1). Pale yellow solid, yield 61%, m.p. 97 °C. IR (nujol): 1715 (C=O ketone), 1643 (C=O amide), and 1592 (C-C aromatic) cm^−1^. ^1^H NMR (CDCl_3_) *δ*: 2.54 (t, 4H, *J* = 6.3 Hz, **H3** and **H5** piperidone), 3.92, 3.91 (s, 6H, C**H_3_**O-), 3.97 (t, 4H, *J* = 6.3 Hz, **H2** and **H6** piperidone), 5.29 (s, 1H, -NH-), 6.78 (d, 1H, *J* = 15.3 Hz, ArHC=C**H**-), 6.87 (d, 1H, *J* = 8.3 Hz, aromatic phenyl **H4**), 7.04 (d, 1H, *J* = 1.8 Hz, aromatic phenyl **H2**), 7.13 (dd, 1H, *J* = 8.3, 1.8 Hz, aromatic phenyl **H6**), and 7.69 (d, 1H, *J* = 15.3 Hz, Ar**H**C=CH-) [34].

*(E)-1-(3-(3,5-di-tert-butyl-4-hydroxyphenyl)acryloyl)piperidin-4-one,* **10**. 

Flash chromatography (petroleum ether/ethyl acetate, 3/1). Pale yellow solid, yield 64%, m.p. 139 °C. IR (nujol): 3494 (O-H), 1730 (C=O ketone), 1644 (C=O amide), 1590, and 1573 (C-C aromatic) cm^−1^. ^1^H NMR (CDCl_3_) *δ*: 1.46 (s, 18H, (C**H_3_**)**_3_**C-), 2.54 (t, 4H, *J* = 6.3 Hz, **H3** and **H5** piperidone), 3.97 (s, 4H, **H2** and **H6** piperidone), 5.30 (s, 1H, -NH-), 5.49 (s, 1H, -O**H**), 6.74 (d, 1H, *J* = 15.3 Hz, ArHC=C**H**-), 7.37 (s, 2H, aromatic phenyl **H2** and **H6**), and 7.71 (d, 1H, *J* = 15.3 Hz, Ar**H**C=CH-). Anal. Calcd for C_22_H_31_ΝO_3_: C, 73.91; H, 8.74; Ν, 3.92. Found: C, 73.87; H, 8.69; Ν, 3.98.

#### 3.2.4. General Method for the Synthesis of Compounds **11**–**13**

In a solution of the corresponding acid (1 mmol) in dichloromethane (CH_2_Cl_2_) or tetrahydrofuran in the case of p-coumaric acid, 4-dimethylaminopyridine (DMAP, 0.5 mmol), 1-methylpiperazine (1 mmol), and *N,N*’-dicyclohexylcarbodiimide (DCC, 1.2 mmol) were added. The reaction mixture was stirred for 24 h at room temperature under nitrogen. After filtration, the solution was washed with aqueous NaHCO_3_ 5% solution, saturated NaCl solution, and dried over Κ_2_CO_3_. The final compounds were isolated with flash chromatography using ethyl acetate, methanol, and triethylamine as eluents in proportion 10:0.5:0.03.

*(E)-3-(4-hydroxyphenyl)-1-(4-methylpiperazin-1-yl)prop-2-en-1-one,* **11**. 

Pale red solid, yield 82%, m.p. 146–148 °C. IR (nujol): 3400–3200 (O-H), 1643 (C=O amide), and 1584 (C-C aromatic) cm^−1^. ^1^H NMR (CDCl_3_) *δ*: 2.34 (s, 3H, C**H_3_**N-), 2.49–2.46 (m, 4H, Ν-methylpiperazine **H2** and **H6**), 3.81–3.64 (m, 4H, Ν-methylpiperazine **H3** and **H5**), 6.69 (d, 1H, *J* = 15.3 Hz, ArHC=C**H**-), 6.84 (d, 2H, *J* = 8.6 Hz, aromatic phenyl **H3** and **H5**), 7.36 (d, 2H, *J* = 8.6 Hz, aromatic phenyl **H4** and **H6**), and 7.61 (d, 1H, *J* = 15.3 Hz, Ar**H**C=CH-) [31].

*(E)-3-(3,4-dimethoxyphenyl)-1-(4-methylpiperazin-1-yl)prop-2-en-1-one,* **12**. 

Pale yellow liquid, yield 77%. IR (nujol): 1638 (C=O amide) and 1584 (C-C aromatic) cm^−1^. ^1^H NMR (CDCl_3_) *δ*: 2.33 (s, 3H, C**H_3_**N-), 2.51–2.42 (m, 4H, Ν-methylpiperazine **H2** and **H6**), 3.81–3.64 (m, 4H, Ν-methylpiperazine **H3** and **H5**), 3.91, 3.89 (s, 6H, C**H_3_**O-), 6.71 (d, 1H, *J* = 15.3 Hz, ArHC=C**H**-), 6.85 (d, 1H, *J* = 8.3 Hz, aromatic **H5**), 7.02 (d, 1H, *J* = 1.9 Hz, aromatic **H2**), 7.10 (dd, 1H, *J* = 8.3, 1.9 Hz, aromatic **H6**), and 7.61 (d, 1H, *J* = 15.3 Hz, Ar**H**C=CH-). Anal. Calcd for C_1622_H_17_Ν_2_O_3_: C, 66.18; H, 7.64; Ν, 9.65. Found: C, 66.31; H, 7.59; Ν, 9.57.

*(E)-3-(3,5-di-tert-butyl-4-hydroxyphenyl)-1-(4-methylpiperazin-1-yl)prop-2-en-1-one,* **13**. 

Pale orange solid, yield 78%, m.p. 170 °C. IR (nujol): 3218 (O-H), 1642 (C=O amide), and 1592 (C-C aromatic) cm^−1^. ^1^H NMR (CDCl_3_) *δ*: 1.45 (s, 18H, (C**H_3_**)**_3_**C-), 2.32 (s, 3H, C**H_3_**N-), 2.46–2.41 (m, 4H, Ν-methylpiperazine **H2** and **H6**), 3.71 (d, 4H, *J* = 27.4 Hz, Ν-methylpiperazine **H3** and **H5**), 5.44 (s, 1H, -O**H**), 6.67 (d, 1H, *J* = 15.3 Hz, ArHC=C**H**-), 7.34 (s, 2H, aromatic phenyl **H2** and **H6**), and 7.63 (d, 1H, *J* = 15.3 Hz, Ar**H**C=CH-). Anal. Calcd for C_22_H_34_Ν_2_O_2_: C, 73.70; H, 9.56; Ν, 7.81. Found: C, 73.65; H, 9.62; Ν, 7.86.

### 3.3. Effect on Lipid Peroxidation

A hepatic microsomal fraction from untreated rats was prepared. The incubation mixture contained heat-inactivated (at 90 °C for 90 s) microsomal fraction, corresponding to 2.5 mg protein/mL (final concentration) or 4 mM fatty acid residues, ascorbic acid (0.2 mM) in Tris–HCl/KCl buffer (50/150 mM), and the test compounds dissolved in dimethylsulfoxide (dimethylsulfoxide alone was used for control groups). The peroxidation reaction was initiated by the addition of a freshly prepared FeSO_4_ solution (10 μM), and the mixture was incubated at 37 °C. Aliquots (0.3 mL) were taken at various time intervals for 45 min. Lipid peroxidation was assessed spectrophotometrically (535 against 600 nm) by the determination of 2-thiobarbituric acid reactive material. All compounds, as well as dimethylsulfoxide, were tested and found not to interfere with the assay [25].

### 3.4. Interaction with the Stable Radical 1,1-Diphenyl-2-picrylhydrazyl (DPPH)

Compounds (in absolute ethanol, final concentrations 25–200 μM) were added to an equal volume of ethanolic solution of DPPH (final concentration 200 μM) at room temperature (22 ± 2 °C). Ethanol without the addition of a compound was used in the case of the control. Absorbance (517 nm) was recorded every 5 min for 30 min in total [25].

### 3.5. Effect on Plasma Total Cholesterol, Triglyceride and LDL-Cholesterol Levels

A solution of Triton WR 1339 (tyloxapol) in saline was administered i.p. (200 mg/kg) to male rats once, and half an hour later, the examined compound (0.15 mmol/kg) (suspended in water with a few drops of Tween 80) was administered i.p. once. Control groups were treated with saline only instead of suspension of the compound. After 24 h, blood was taken from the aorta under hexobarbital anesthesia and used for the determination of plasma total cholesterol (TC) and triglyceride (TG) concentrations, using commercial kits, against standard solutions [38]. Rats were kept on Purina laboratory chow and tap water *ad libitum* throughout the experiment until hexobarbital anesthesia took place.

## 4. Conclusions

In the present study, substituted 3-phenylacrylic acid derivatives with morpholine, 3-hydroxymethylpyridine, 4-piperidone, and 4-methylpiperazine were designed to attain antioxidant and hypolipidemic properties for the prevention or restoration of pathological conditions implicating degeneration and oxidative stress.

The incorporation of (*E*)-3-(3,5-di-*tert*-butyl-4-hydroxyphenyl)acrylic acid provided increased and comparable antioxidant and reducing capacity to Trolox with hypocholesterolemic and hypotriglyceridemic activities. Sinapic acid derivative (compound **6**) was more active than the ferulic derivative (compound **5**) both as an antioxidant and hypolipidemic. However, the interrelation between these activities is not parallel in all the cases, since compounds **11** and **12**, with no antioxidant potency, had increased hypolipidemic activity, whilst compound **8**, with increased antioxidant activity, was a significantly lower hypolipidemic than the other two. Compounds **4** and **13** were the most active antioxidants and were among the four most active hypocholesterolemics and the three most active hypotriglyceridemic derivatives, possessing, in a high degree, both the activities they were tested for. In this research, most of the described compounds, especially compounds **4** and **13**, offer pleiotropic activity. If we take into account that beneficial treatment of diseases, which include oxidative stress and lipid deregulation, may be addressed with lipid-lowering antioxidants, then derivatives, like those described in this project, may contribute in this direction, overcoming potential drug–drug interactions and decreasing the polypharmacy phenomena and adverse effects that may derive from multi-drug treatment.

## Figures and Tables

**Table 1:** Effect of the compounds **4**, **5**, **6**, **8**, **9**, **10**, **13**, and **Trolox** on rat hepatic microsomal membrane lipid peroxidation.

Compound	Lipid Peroxidation Inhibition: IC_50_ (μΜ) ^a^
**4**	42
**5**	545
**6**	189
**8**	42
**9**	205
**10**	37
**13**	41
**Trolox**	25

^a^ After 45 min of incubation, **Trolox**: 6-hydroxy-2,5,7,8-tetramethylchroman-2-carboxylic acid. All determinations were performed in triplicate, and standard deviation was always within ±10% of the mean value.

**Table 2:** Interaction of compounds **2**, **4**, **5**, **6**, **8**, **10**, **13**, and **Trolox** at various concentrations, with DPPH (200 μΜ) ^a^.

Compound	Percent Interaction				IC_50_ (μΜ) ^b^
	200 μΜ	100 μΜ	50 μΜ	25 μΜ	
**2**	90.70	80.68	74.19	34.74	31.83
**4**	95.63	81.88	78.20	32.92	31.75
**5**	65.00	62.27	27.16	0.00	99.66
**6**	71.93	69.56	27.56	0.00	87.07
**8**	95.08	81.41	70.11	34.01	34.48
**10**	90.48	82.50	69.38	33.65	34.51
**13**	92.96	86.15	73.57	32.48	33.05
**Trolox**	98.00	92.00	38.00	22.00	52.01

^a^ After 30 min of incubation. ^b^ The concentration of the compounds that cause 50% interaction with DPPH (200 μΜ). **Trolox**: 6-hydroxy-2,5,7,8-tetramethylchroman-2-carboxylic acid. All determinations were performed in triplicate, and standard deviation was always within ±10% of the mean value.

**Table 3:** Effect of the compounds **1**–**13** and simvastatin on Triton WR1339 (tyloxapol)-induced hyperlipidemia and the calculated lipophilicity (clog*P*).

Compound	% Reduction		clog*P* ^c^
	TC ^a^	TG ^b^	
**1**	8.4 ^ns^	8.4 ^ns^	0.95
**2**	34.1 ***	33.0 ***	0.73
**3**	39.9 ***	44.6 ***	1.42
**4**	58.6 ***	64.2 ***	4.55
**5**	35.1 **	44.6 ***	1.92
**6**	50.8 ***	69.5 ***	1.70
**7**	33.0 ***	35.1 ***	2.39
**8**	40.2 *	35.1 ***	5.52
**9**	38.9 ***	24.6 *	1.36
**10**	51.1 ***	35.7 *	4.49
**11**	64.3 ***	51.5 ***	1.66
**12**	59.8 ***	61.9 ***	1.86
**13**	59.3 ***	69.4 ***	5.11
**Simvastatin**	73 ***	-	4.48

^a^ TC: total cholesterol; ^b^ TG: triglycerides; ^c^ calculated with ChemBioDraw Ultra. Tyloxapol: 200 mg/kg, *i.p*.; compounds and simvastatin: 150 μmol/kg, *i.p.* -: not statistically significant result. Significant difference from hyperlipidemic control: ^ns^ not statistically significant, * *p* < 0.05, ** *p* < 0.005, and *** *p* < 0.0005 (Student’s *t*-test).

## Data Availability

The data presented in this study are available on request from the corresponding author.
